# Establishing reference intervals for von Willebrand factor multimers

**DOI:** 10.5937/jomb0-31941

**Published:** 2022-02-02

**Authors:** Marika Pikta, Marc Vasse, Kristi J. Smock, Karen A. Moser, Dievoet Marie-Astrid van, Sandra Lejniece, Timea Szanto, Hector Bautista, George Nouadje, Valdas Banys

**Affiliations:** 1 North Estonia Medical Centre, Department of Laboratory Medicine, Tallinn, Estonia; 2 Tallinn University of Technology, Department of Health Technologies, Tallinn, Estonia; 3 Foch Hospital, Department of Biology & UMR INSERM 1176, Suresnes, France; 4 University of Utah, School of Medicine, Department of Pathology, Salt Lake City, UT, USA; 5 ARUP Institute for Clinical and Experimental Pathology®, Salt Lake City, UT, USA; 6 Cliniques Universitaires Saint-Luc, Laboratory Department, Bruxelles, Belgium; 7 Riga Stradins University, Riga, Latvia; 8 Riga East University Hospital, Chemotherapy and Hematology Clinic, Riga, Latvia; 9 Helsinki University Hospital Comprehensive Cancer Center and University of Helsinki, Department of Hematology, Coagulation Disorders Unit, Helsinki, Finland; 10 Sebia, Research and Developments Department, Lisses - Evry Cedex, France; 11 Vilnius University, Faculty of Medicine, Institute of Biomedical Sciences, Department of Physiology, Biochemistry, Microbiology and Laboratory Medicine, Vilnius, Lithuania

**Keywords:** von Willebrand factor, von Willebrand factor multimers, quantitative analysis, reference intervals, von Willeberandov faktor, multimeri von Willebrandovog faktora, kvantitativna analiza, referentni intervali

## Abstract

**Background:**

von Willebrand factor (VWF) multimers (VWF:MM) methodologies are technically difficult, laborious, time consuming, non-standardized and results vary between laboratories. A new semi automated VWF:MM assay is available for routine use (Sebia). Due to lack of reference values for VWF:MM fractions, results interpretation can be challenging in some cases. The aim of this study was to determine reference intervals for low molecular weight (LMWM), intermediate molecular weight (IMWM) and high molecular weight (HMWM) multimers.

**Methods:**

By the international cooperation initiated between 4 countries (Estonia, Latvia, France, and USA) 131 samples of relatively healthy individuals were analyzed for VWF:MM (in total 51 males and 80 non-pregnant females aged 17-69 years). Reference intervals were calculated according to CLSI C28-A3 standard.

**Results:**

The proposed reference intervals for VWF:MM were calculated for LMWM 10.4-22.5%, IMWM 22.6-37.6%, HMWM 45.6-66.6%. Age related differences were seen in IMWM and HMWM (p<0.001 and 0.038). There was no gender related difference observed. Geographically LMWM results of France were different from the other regions (p<0.05).

**Conclusions:**

Quantification of VWF:MM fractions, in addition to qualitative assessment of VWF:MM patterns, has the potential to aid in differential diagnosis of von Willebrand disease (VWD) subtypes. The reference values calculated in this study can be used in future research to establish clinical decision limits.

## Abstract (sr)

**Uvod:**

Metodologija multimera (VWF:MM) von Willebrand faktora (VWF) tehnički je teška, naporna, digotrajna, nestandardizovana i rezultati se razlikuju u različitim laboratorijama. Novi poluautomatski VWF:MM test (Sebia) dostupan je za rutinsku upotrebu. Zbog nedostatka referentnih vrednosti za VWF:MM frakcije, tumačenje rezultata može u nekim slučajevima biti izazovno. Cilj ove studije bio je da se odrede referentni intervali za multimere niske molekularne mase (LMWM), srednje molekularne mase (IMWM) i visoke molekularne težine (HMWM).

**Metode:**

Međunarodnom saradnjom započetom između 4 zemlje (Estonija, Letonija, Francuska i SAD) 131 uzorak relativno zdravih pojedinaca analiziran je na VWF:MM (ukupno 51 muškarac i 80 žena koje nisu bile trudne u dobi od 17-69 godina). Referentni intervali su izračunati prema CLSI C28-A3 standardu.

**Rezultati:**

Predloženi referentni intervali za VWF:MM izračunati su za LMWM 10,4-22,5%, IMWM 22,6-37,6% i HMWM 45,6-66,6%. Starosne razlike su primećene u IMWM i HMWM (p < 0,001 i 0,038). Nije uočena razlika vezana za pol. Geografski rezultati LMWM iz Francuske bili su različiti od ostalih regiona (p < 0,05).

**Zaključak:**

Kvantifikovanje frakcija VWF:MM, pored kvalitativne procene VWF:MM uzoraka, može da pomogne i u diferencijalnoj dijagnozi podtipova von Willebrandove (VWD) bolesti. Referentne vrednosti izračunate u ovoj studiji mogu se koristiti u budućim istraživanjima za utvrđivanje granica kliničkih odluka.

## Introduction

Von Willebrand disease (VWD) is the most common inherited bleeding disorder with an approximate prevalence of about 1-2% in the general population [1] [2] [3], although the true incidence is unknown [4]. VWF plays an important role in regulation of normal hemostasis and facilitates progression of bleeding or thrombotic disorders with platelet and endothelial dysfunction [5] [6]. VWD arises due to structural and/or quantitative abnormalities of von Willebrand factor (VWF), a large multimeric glycoprotein with adhesive functions through binding to FVIII, to platelet surface glycoproteins, and to constituents of subendothelial connective tissue [5] [6] [7].

VWD is classified into 3 main types: type 1, a partial quantitative deficiency; type 2, a qualitative defect that is further subdivided into 4 categories, 2A, 2B, 2N, and 2M; and type 3, a complete absence of VWF [1]. Correct classification of the type/subtype of the VWD is important in patients' management and the therapeutic approach [1].

As VWF has diverse functions, laboratory testing for VWD and other VWF-related disorders (i.e., thrombotic thrombocytopenic purpura [8] or a variety of cardiac lesions that result in clearance of larger multimers, such as aortic regurgitation, mitral insufficiency, and hypertrophic cardiomyopathy [9]) require complex laboratory assessment [3] [10]. The first-line tests typically include evaluation of VWF antigen (VWF:Ag), different VWF activity (VWF:Ac) assays (e.g. ristocetin cofactor assay (VWF:RCo), VWF activity measured as VWF binding to the glycoprotein Ib (GPIb) receptor on the platelet surface (VWF:GPIbM), collagen binding (VWF:CB) etc.) and factor VIII activity (FVIII:C) [4].

VWF multimeric assay is a second-line analysis used in the diagnosis and classification of different VWD subtypes [11]. VWF circulates in plasma as low, intermediate, and high molecular weight (LMWM, IMWM, and HMWM, respectively) multimers [12] [13]. The absence of HMWM is the cardinal feature that distinguishes type 1 from type 2A and 2B VWD, whereas the different subtypes of type 2 VWD can be differentiated by more subtle alterations of the inner structure of smaller multimers [4] [10] [11].

Historically, VWF multimers are analyzed by inhouse developed electrophoresis techniques and densitometric analysis of Western blots [7] [14]. These methodologies are technically difficult, laborious, time consuming and non-standardized [2] [12]. The development of a relatively rapid semi-automated commercial VWF multimer kit assay (Hydragel 5/Hydragel 11 von Willebrand multimers, Sebia, France) may represent a first step toward standardization. This method was already shown to provide adequate information for characterization and classification of congenital VWD subtypes [12] [14] [15]. Moreover, results correlate with the clinical status, diagnosis of inherited or acquired VWD, if used and interpreted by experienced professionals [12] [14].

In addition to qualitative interpretation of multimer patterns, the Sebia PHORESIS software allows quantification of VWF:MM band patterns, and calculation of the percentage values of each molecular weight multimer fraction. Quantitative multimer analysis might be needed for the detection of subtle abnormalities and changes following therapeutic interventions [7] [16]. Due to lack of reference values for VWF:MM fractions, result interpretation can be challenging in some cases.

Thus, in the present study we used densitometry to determine reference intervals for LMWM, IMWM and HMWM fractions.

## Materials and Methods

### Study subjects

To collect a larger sample size an international cooperation was initiated between 4 countries (Estonia, Latvia, France, and USA). The list of participating institutions were as follows: L1 (two institutions from Baltic countries: L1A - Laboratory of North Estonia Medical Centre, Tallinn Estonia; L1B - Riga East University Hospital, Riga, Latvia), L2 (Department of Biology, Foch Hospital, Suresnes, France), and L3 (University of Utah/ARUP Laboratories, Salt Lake City, Utah, United States). Both Estonian and Latvian samples were analyzed in the Laboratory of North Estonia Medical Centre, thus accounted as one group L1.

In total 134 healthy volunteers were recruited for this study, but after outlier exclusion 131 samples were analyzed: 51 males and 80 non-pregnant females aged 17-69 years.

Acceptance criteria: no history of hemorrhagic episodes; no usage of any interfering medication for at least 10 days before blood collection; normal VWF results (VWF:Ag; VWF:Ac - VWF:GPIbM (L1), VWF: GPIbR (L2) and VWF:RCo (L3); VWF:Ac/VWF:Ag ratio); written consent provided. Blood donor plasmas were not used because the questionnaire for blood donors do not include information regarding family bleeding history, individual mild bleeding episodes and are not screened for VWD routinely. The study was performed according to the Declaration of Helsinki and was approved by appropriate local or national ethical committees or local Institutional Review Board at each institution.

### Sample collection and specimen processing

Samples for the reference interval studies were collected from apparently healthy individuals according to the participating institutions' locally approved venous blood sampling procedures and in concordance with ethical laws of each participating country. Briefly, peripheral venous blood specimens were collected into light blue-top vacuum tubes [3.2% sodium citrate tubes (BD Vacutainer, L1A, L3 or Sarstedt, L2) or 3.8% NC Buffered Citrate (Vacutest KIMA srl, L1B)], centrifuged (within 2 hours after sampling) at a speed and time required to consistently produce platelet-poor plasma (residual platelet count less than 10 x10^9^ L):

L1A - 1500 g for 15 minutes at room temperature

L2B - 1500 g for 15 minutes at room temperature, aliquoted, stored frozen at -70°C and transported on dry ice to L1A

L2 - 2000 g for 15 minutes at 15°C (twice)

L3 - 1700 g for 15 minutes at room temperature

Samples were aliquoted and stored frozen (at least -20°C) until testing (within 30 days). Aliquots were thawed in a water bath (+37°C) for 5 minutes and mixed well before testing.

### VWF multimers method and densitometry

The VWF multimers method, developed by Sebia (France), is described in detail elsewhere [3] [4]. It was used by the participating laboratories without deviation from the original Sebia assay protocol. In brief, citrated plasma samples were analyzed on the Hydrasys 2 instrument (Sebia, France) with ready to use SDS agarose gels (Hydragel 5 von Willebrand multimers, Sebia). Densitometry of VWF multimer patterns was carried out with a transmission scanner (Sebia Gelscan Instrument) which allows scanning and data storage of the results. Data acquisition is performed by a bidimensional calibrated CCD sensor. The instrument, when connected to a PC with the Sebia PHORESIS software, allowed the operator to display the gel images, curves, curves overlapping, and quantification of multimer band patterns according to the manufacturer recommendation (LMWM 1-3 bands; IMWM 4-7 bands; and HMWM 8^th^ band and above).

The percentage values of each molecular weight multimer fraction was provided by the software. The calculation was made by applying the ratio of the area of each fraction and the total area under the curve. The multimer patterns of the plasma samples studied were, if necessary, compared with the reference pool pattern analyzed on the same gel. The total area under the curve of each sample was directly proportional to the amount of antigen (VWF:Ag).

### Statistical analysis

All statistical analysis was performed with MedCalc® software (MedCalc Software, Belgium) version 18.11.6. and IBM SPSS statistics version 23. Descriptive statistics was used to analyze demographic data and laboratory characteristics. The data was analyzed according to age, gender and geographic location. The results were expressed as median (interquartile range [IQR]). The difference between variables was tested using the Mann-Whitney test. P values of <0.05 were considered statistically significant.

Reference intervals were established using a robust method following CLSI C28-A3 standard to calculate the 2.5^th^ and 97.5^th^ percentiles and associated 90% confidence intervals (CI) for each VWF multimeric fraction. Data distributions were tested for normality by Shapiro-Wilk test. Outlier detection was performed by Grubs double sided and Tukey methods.

## Results

### Study subjects

Data and samples were collected from 131 healthy volunteers (51 males and 80 non-pregnant females), from Baltic Region (L1), France (L2) and United States (L3). The demographic characteristics and laboratory findings are summarized in Table 1.

**Table 1:** Characteristics of study groups and corresponding results of VWF:MM fractions

	L1 (n=31)	L2 (n=64)	L3 (n=36)
Age range (years)	18–69	17-62	19–61
Age, median (IQR)	34 (23–46)	40.5 (30.3 –51.8)	30 (24.3–36.0)
males/females	7/24	27/37	17/19
LMWM, % median (IQR)	15 (12.7–17.2)	16.1 (14.5–19.1)	14 (12.4–16.0)
LMWM lowest / highest value	9.8–23.0	10.7–23.3	9.7–19.9
IMWM, % median (IQR)	29.2 (26.7–31.2)	29 (27.2–30.6)	30.7 (26.3–34.2)
IMWM lowest / highest value	22.8–36.4	21.4–35.8	21.3–38.6
HMWM, % median (IQR)	55.4 (51.1–60.2)	54.5 (52.2–58.1)	55.9 (51.3–59.6)
HMWM lowest / highest value	43.2–66.2	45.1–65.2	44.4–68.2

**Figure 1:**
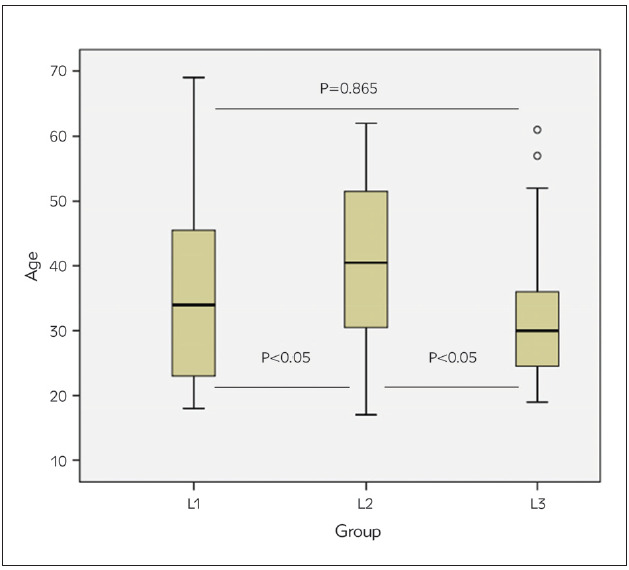
Age differences between subjects of different geographical locations L1, samples from Baltic region; L2, samples from France; L3, samples from United States

Participants' age was between 17 and 69 years. Subjects from L3 were younger than from L1 and L2: medians (IQR) were 30 (24.3-36.0), 34 and 40.5 (30.3-51.8), respectively. As presented in Figure 1, there was no significant difference in age between L1 and L3 (P=0.865), but the differences between L2 vs L1 and L2 vs L3 were statistically significant (p<0.05).

To assess possible differences in VWF multimers fractions data from the 3 participating regions was compared.

### Age related difference in VWF multimers fractions

**Figure 2:**
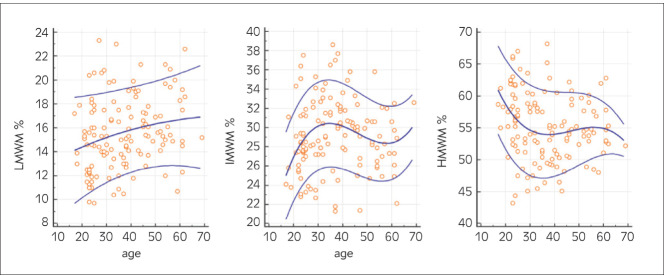
Age-related differences of LMWM, IMWM and HMWM in study population LMWM, low-molecular-weight multimers; IMWM, intermediate-molecular-weight multimers HMWM, high-molecular-weight multimers<br>Blue lines represent 0.1, 0.5 and 0.9 centiles

VWF multimers patterns were analyzed for agerelated differences and are shown in Figure 2. Visually LMWM tend to increase with increasing age, although changes are not statistically significant. IMWM variations were found to be statistically significant (P<0.001), but values fluctuate with two intervals with increasing values, and one shift of decreasing values. HMWM tend to decrease with increasing age, and this finding is statistically significant (P=0.038).

### Gender related difference in VWF multimers fractions

**Figure 3:**
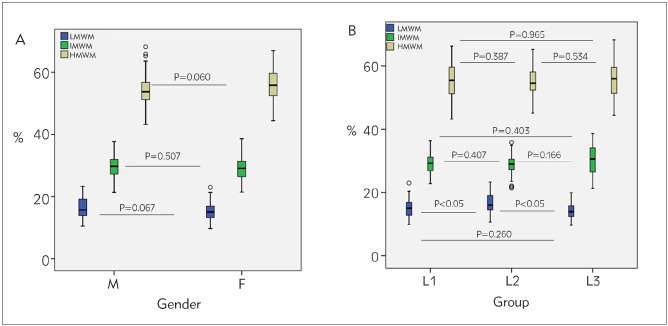
Differences of LMWM, IMWM and HMWM percentage values by gender (A) and between geographical locations (B) L1, samples from Baltic region; L2, samples from France; L3, samples from United States; LMWM, low-molecular-weight multimers; IMWM, intermediate-molecular-weight multimers HMWM, high-molecular-weight multimers

As shown in Figure 3A, there was no significant difference between males and females in VWF multimers structure: LMWM (P=0.067), IMWM (P=0.507), HMWM (P=0.060).

### Geographical locations related difference in VWF multimers fractions

Table 1 and Figure 3B summarize the results of the VWF structure related parameters.

The LMWM were higher in group L2 (16.1 [14.5-19.1]) than in group L1 (15 [12.7-7.2]) and group L3 (14 [12.4-16.0]). The differences between L2 vs L1 and L2 vs L3 were statistically significant (p<0.05) but clinically irrelevant, difference between L1 vs L3 was insignificant (P=0.260). There was no significant difference in IMWM and HMWM between geographical locations.

### Calculation of reference intervals

Values of the three testing locations for the LMWM, IMWM and HMWM were distributed normally, thus reference values were calculated based on a normal distribution.

The proposed reference intervals for VWF:MM are presented in Table 2.

**Table 2:** Proposed reference intervals for VWF:MM

	Low<br>Molecular<br>Weight	Intermediate<br>Molecular<br>Weight	High<br>Molecular<br>Weight
Lower limit, %<br>[90% CI]	10.4<br>[9.9–11.0]	22.6<br>[21.8–23.3]	45.6<br>[44.5–46.7]
Upper limit, %<br>[90% CI]	22.5<br>[21.5–23.5]	37.6<br>[36.4–38.7]	66.6<br>[65.1–68.0]
			

## Discussion

VWF multimeric analysis is essential for diagnosis and subtyping of VWD and acquired von Willebrand syndrome (AVWS) [5] [9] [17] [18] [19]. There is still a need for interlaboratory standardization of this method. Indeed, interlaboratory comparability and reproducibility of this analysis are insufficient due to the predominant use of locally developed VWF multimer methods by laboratories worldwide [20] [21]. The new semi-automated VWF multimer technique can help in standardization [22]: it helps to reduce the interlaboratory variability and the variability between different measurement runs. Densitometry could contribute to its standardization by offering a reproducible quantification and additional visualization of VWF multimer patterns and permitting a precise quantitative comparison of sample patterns with those of a reference plasma curve [23].

Several independent investigators have previously reported on the analytical performance evaluation of the new Sebia technique with either 5-gel and 11-gel formats [3] [12] [14] [15] [18] [23] [24] [25]. Details of analytical performance of the Sebia method are beyond the scope of our current study. In brief, this new assay provides a clear pattern of VWF multimer distribution on the gels and densitometry scans. It demonstrates acceptable performance results and has the major advantage of being performed within one working day.

In published data for evaluation of the accuracy of the new Sebia assay researchers have used different approaches. They have compared plasma samples from patients presenting with different types of VWD with samples from healthy volunteers [24], commercial Standard Human Plasma [25], donors and commercial frozen normal donor plasmas [14]. Reference intervals were not originally defined by the manufacturer. Due to lack of reference values for VWF:MM fractions, results interpretation can be challenging in some cases. HMWM have the greatest role in VWF functional activity [13], therefore reference intervals for HMWM are most important in clinical decision making.

In 2018, Bowyer et al. [14] investigated multimeric patterns in 51 samples collected from healthy volunteers and using commercial frozen normal donor plasma (Cryocheck; Precision Biologic, Halifax, NS, Canada). In this study ranges for HMWM varied 35-58.5%, but authors noted that Gaussian distribution was not observed for HMWM. Importantly, the storage condition for the commercial Cryocheck Normal Donor Set is at -40 to -80°C. Storage and transport issues that allowed plasmas to reach temperatures outside of this range potentially could have affected the establishment of HMWM lower intervals using this donor set.

A group of researchers from Belgium [24] has calculated normal reference intervals for VWF multimers fractions using samples from 40 healthy volunteers. They have reported intervals for HMWM as 40.8-63.2%.

The intervals determined in these previous studies were similar to our results, but they were calculated using a relatively low powered sample size. According to the CLSI guidelines C28-A3 [26], the sample size can be considered to be representative when it is larger than 120, therefore in the current study we established the reference intervals of LMWM, IMWM and HMWM fractions in 131 relatively healthy adults, in order to obtain a more acccurate result.

An interesting finding was the relationship of certain multimer fractions with the age of study individuals. The tendency of LMWM to increase and HMWM to decrease with increased age is seen in our data. Meanwhile, IMWM values are variable during adult life. Nevertheless, definitive conclusions cannot be made due to the small sample size of the study. Discovered tendencies, especially the tendency of HMWM to decrease with increasing age, could potentially be analyzed in detail in future larger studies.

It should be noted that multimer fraction separation and their percentage values calculation is based on the scanned gel and are not directly measured quantitatively, thus an interpretation of »gray zone« should be considered in future studies evaluating clinical decision making possibilities.

To conclude, the quantification of VWF:MM fractions is an additional valuable tool to supplement the qualitative visual assessment of VWF:MM patterns. It potentially has the value to aid in differential diagnosis of VWD and AVWS subtypes. The reference values calculated in this study can be used in future research to establish clinical decision limits.

## Dodatak

### Acknowledgments

We are grateful to Sebia (France) for the funding of this project. We would like to thank all the medical/laboratory staff and the volunteers who contributed to this study. The preliminary results of this study were discussed at IFCC C-RIDL (Scientific Committee of IFCC on Reference Intervals and Decision Limits) closed meeting during Euro-medlab2019 Barcelona congress and presented atthe ISTH 2020 Virtual Congress (abstract/e-poster).

### Conflict of interest statement

All the authors declare that they have no conflict of interest in this work.
